# Extracellular vesicles produced by human and animal *Staphylococcus aureus* strains share a highly conserved core proteome

**DOI:** 10.1038/s41598-020-64952-y

**Published:** 2020-05-21

**Authors:** Natayme Rocha Tartaglia, Aurélie Nicolas, Vinícius de Rezende Rodovalho, Brenda Silva Rosa da Luz, Valérie Briard-Bion, Zuzana Krupova, Anne Thierry, François Coste, Agnes Burel, Patrice Martin, Julien Jardin, Vasco Azevedo, Yves Le Loir, Eric Guédon

**Affiliations:** 1 INRAE, Institut Agro, STLO, F-35000 Rennes, France; 2 Institute of Biological Sciences, Federal University of Minas Gerais, Minas Gerais Belo Horizonte, Brazil; 3 Excilone, F-78990 Elancourt, France; 4 Univ Rennes, Inria, CNRS, IRISA, Rennes, France; 5 Univ Rennes, CNRS, Inserm, BIOSIT - UMS 3480, US_S 018, F-35000, Rennes, France; 6 INRAE, Université Paris-Saclay, AgroParisTech, UMR GABI, F-78350 Jouy-en-Josas, France

**Keywords:** Pathogens, Pathogenesis

## Abstract

*Staphylococcus aureus* is an important opportunistic pathogen of humans and animals. It produces extracellular vesicles (EVs) that are involved in cellular communication and enable inter-kingdom crosstalk, the delivery of virulence factors and modulation of the host immune response. The protein content of EVs determines their biological functions. Clarifying which proteins are selected, and how, is of crucial value to understanding the role of EVs in pathogenesis and the development of molecular delivery systems. Here, we postulated that *S. aureus* EVs share a common proteome containing components involved in cargo sorting. The EV proteomes of five *S. aureus* strains originating from human, bovine, and ovine hosts were characterised. The clustering of EV proteomes reflected the diversity of the producing strains. A total of 253 proteins were identified, 119 of which composed a core EV proteome with functions in bacterial survival, pathogenesis, and putatively in EV biology. We also identified features in the sequences of EV proteins and the corresponding genes that could account for their packaging into EVs. Our findings corroborate the hypothesis of a selective sorting of proteins into EVs and offer new perspectives concerning the roles of EVs in *S. aureus* pathogenesis in specific host niches.

## Introduction

*Staphylococcus aureus* is a Gram-positive opportunistic pathogen that causes a broad spectrum of infections in humans and animals. In humans, these diseases range from superficial skin and soft tissue infections to life-threatening conditions that require hospitalisation and extensive medical support^1,2^. This bacterium is also one of the main causative agents of nosocomial infections. In animals, *S. aureus* is notably responsible for ruminant mastitis, an inflammation of the mammary glands that dramatically affects animal health and welfare, milk quality and the economics of milk production^3^. Mastitis is also the principal reason for the use of antibiotics in dairy herds^4^. The wide range of clinical manifestations of *S. aureus* infections is likely associated with its huge arsenal of virulence factors, which include structural components and extracellular factors such as enzymes and toxins^5^. Despite considerable efforts, the precise mechanisms underlying host adaptation, colonisation and interactions are not yet fully understood^6^.

Extracellular vesicles (EVs) are used by many pathogenic bacteria as a secretory route to deliver toxic compounds to infected cells^7,8^. EVs are lipid bilayer nanoparticles that range in size from 20 to 300 nm and are released by almost all cells in all domains of life^9^. In Gram-positive bacteria, they are formed by budding and shedding of the cytoplasmic membrane. They play a pivotal role in cell-to-cell communication through their ability to transport bioactive molecules (proteins, nucleic acids, lipids, metabolites) from donor to recipient cells. The EVs produced by *S. aureus* can mediate the pathogenesis of infection in a variety of ways. They may be cytotoxic to host cells^10–12^, induce the production of cytokines^13–18^, contribute to biofilm formation^19^, mediate antibiotic resistance^20^ or promote an increase in the *in vivo* survival of *S. aureus*^21^. In addition, the immunogenic and adjuvant properties of *S. aureus* EVs can induce a protective response against *S. aureus* infection, thus offering new alternatives for the design of vaccines and the control of infections^15,17,21,22^. The broad spectrum of activities associated with *S. aureus*-derived EVs, as well as their strain-dependency, is mainly related to their protein content^12^. The EV cargo includes a variety of proteins, such as virulence factors and lipoproteins, some of which are known to be potent antigens^12,17,18,21–24^. The EV protein cargo has been shown to vary as a function of environmental conditions^21^ and the producer strains^12,17^. The identification of proteins shared between the EVs of several strains has suggested that protein packaging in EVs results more from a conserved selective mechanism than from a random process^12,18^. However, to date, the molecular mechanisms that drive the recruitment into EVs of these proteins remain unclear, thus limiting our understanding of the pathophysiological relevance of EVs *in vivo* and the development of EV-based applications.

In eukaryotic cells, the components associated with protein packaging into exosomes, a class of eukaryotic EVs, are found in the exosomes produced by most cell types^25,26^. Likewise, the proteins involved in EV formation in *S. aureus* are also found in the EVs they produce^22,23^. We therefore postulated that *S. aureus* strains might share an EV proteome containing essential components for the cargo selection of proteins into EVs. Considering that secreted proteins are key elements in the virulence and host adaptation of *S. aureus*^27^, we suggested that strains isolated from different hosts (human, bovine, ovine) and involved in different types of infection (from mild to severe) offer the best source of diversity to explore cargo selectivity and determine a robust core EV proteome. During this work, we purified EVs released into the culture supernatant of five *S. aureus* strains and identified the whole set of proteins packaged into these EVs. This enabled identification of a core EV proteome. The distribution, functions, physicochemical properties and amino acid composition of the proteins packaged into EVs as a function of different strains were analysed and compared with regard to the host origin of strains, the severity of infections the strains thus produced could cause and the selectivity of protein cargo into EVs.

## Results

### EV production in *S. aureus* strains of human, bovine and ovine origins

**Figure 1:**
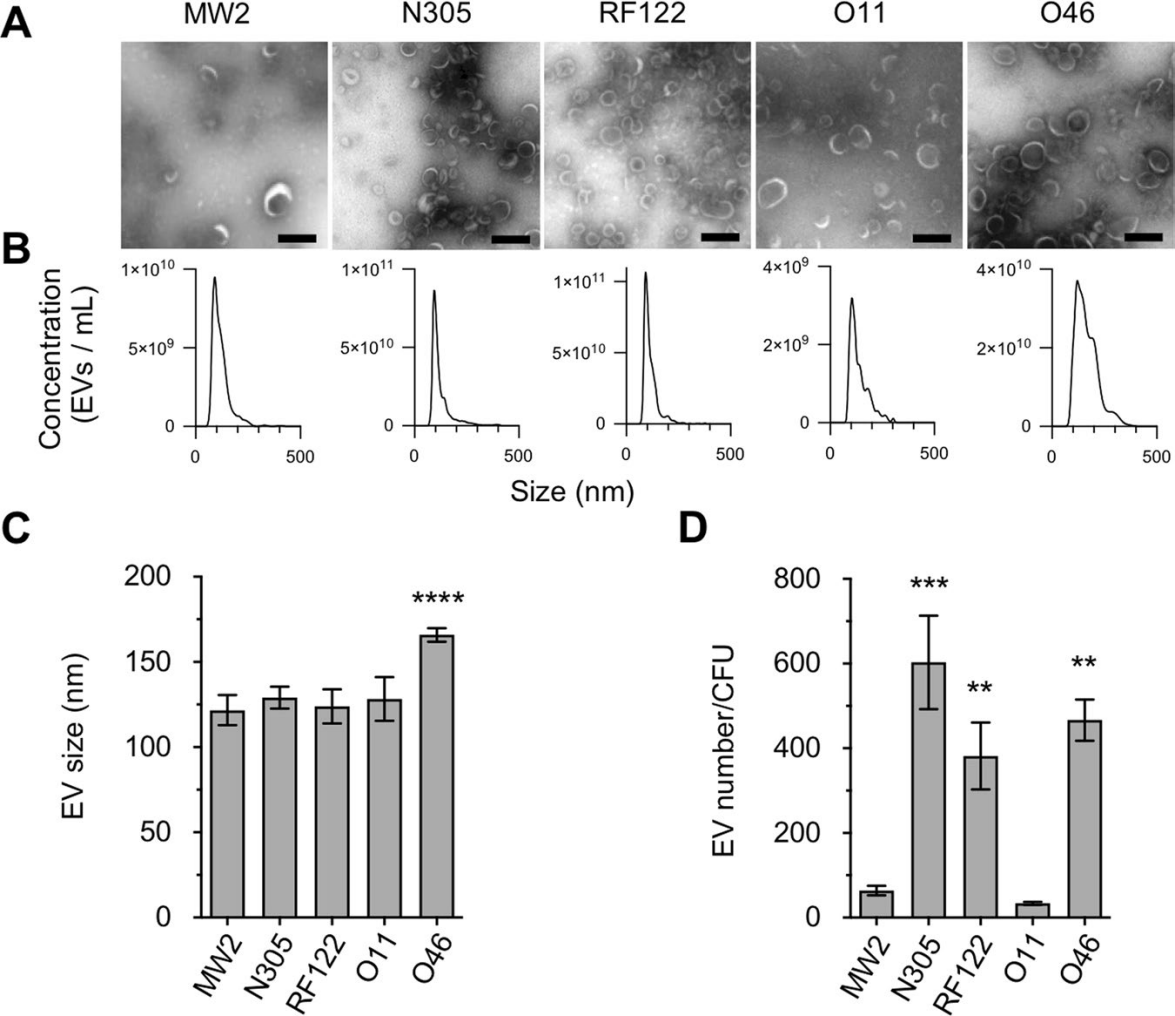
Shape, size and yield of EVs produced by *S. aureus* strains MW2, N305, RF122, O11, and O46. (**A**) Representative electron microscopy images of negatively stained EVs purified from the culture supernatant of *S. aureus* strains MW2, N305, RF122, O11 and O46. Scale bar = 200 nm. (**B**) Representative graphs of the size distribution of EVs purified from culture supernatants of *S. aureus* strains MW2, N305, RF122, O11 and O46. (**C**) Mean size of EVs produced by *S. aureus* strains MW2, N305, RF122, O11 and O46. (**D**) Numbers of EVs produced by *S. aureus* strains MW2, N305, RF122, O11 and O46. Numbers of EVs per milliliter were normalized to numbers of CFU per milliliter calculated from cultures of each strain grown in BHI medium at a time when EVs were recovered. Data are presented as mean + SD of values obtained from three independent EV replicates. Asterisks indicate statistical significance (one-way ANOVA followed by Dunnett’s multiple comparisons test: **P < 0.01; ***P < 0.001) of the mean value for each strain with that of the strain with the lowest mean value (O11).

The ability to produce EVs of five *S. aureus* strains isolated from three types of hosts was investigated. These included two bovine strains, Newbould 305 (hereinafter referred to as N305) and RF122, two ovine strains, *S. aureus* O11 and O46, and the highly virulent human methicillin-resistant *S. aureus* (MRSA) strain MW2. *S. aureus* N305, in which EV production had already been demonstrated, was used as a positive control for the EV preparations^18^. All the *S. aureus* strains used in this study produced EVs with classical features, including a nanoscale size, spherical structure and cup-shaped morphology when visualised using negative staining electron microscopy (Fig. 1A)^28,29^. In addition to EVs, cylindrical (nanotube-like) structures were observed in the *S. aureus* N305, RF122, O11 and O46 samples (Supplementary Fig. S1). The size of the EVs secreted was homogeneous for each strain and similar between strains (120–130 nm), with the exception of strain O46 which secreted significantly larger EVs (170 nm, P < 0.0001) (Fig. 1B,C). Although the growth conditions and EV preparation methods were the same, the number of EVs harvested from the MW2 and O11 culture supernatants was five to ten times lower than that of the other strains, suggesting that the yield of EV production is a strain-specific feature (Fig. 1D).

### Protein composition of *S. aureus* EV proteomes

**Figure 2:**
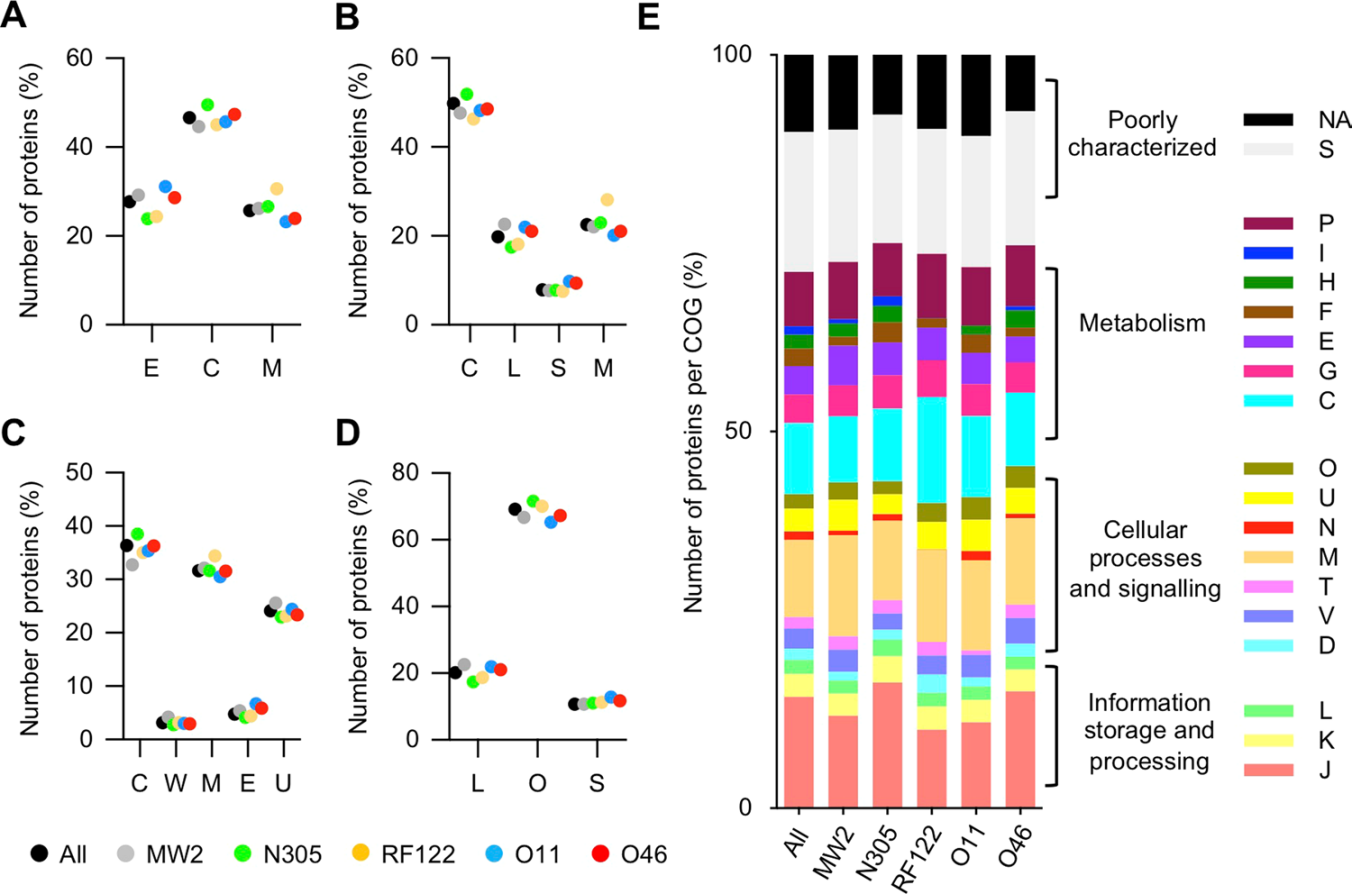
Subcellular localisation and function of *S. aureus* EV proteins. (**A**) CELLO, (**B**) PredLIPO, (**C**) PSORTb, and (**D**) SignalP pipelines were used to predict the subcellular localisation of EV proteins in all the strains tested (pooled dataset; black) and individual strains N305 (green), RF122 (yellow), O11 (blue), O46 (red) and MW2 (grey). C, cytoplasm; E, extracellular; L, lipoprotein; M, membrane; O, other; S, signal peptide I; U, unknown; W, cell-wall. (**E**) Distribution of EV proteins according to their COG classification and strains. C, energy production and conversion; D, cell cycle control/cell division/chromosome partitioning; E, amino acid transport and metabolism; F, nucleotide transport and metabolism; G, carbohydrate transport and metabolism; H, coenzyme transport and metabolism; I, lipid transport and metabolism; J, translation/ribosomal structure and biogenesis; K, transcription; L, replication/recombination/repair; M, cell wall/membrane/envelope biogenesis; N, cell motility; NA, not available; O, posttranslational modification/protein turnover/chaperones; P, inorganic ion transport and metabolism; S, function unknown; T, signal transduction mechanisms; U, intracellular trafficking/secretion/vesicular transport; V, defence mechanisms.

The proteome of the EVs produced by *S. aureus* O11, O46, RF122, and MW2 was determined by Nano LC-ESI-MS/MS analysis on three biological replicates. The dataset was completed by the vesicular proteome of strain N305, which had been obtained during a previous study using the same experimental procedures^18^. A total of 253 proteins associated with *S. aureus* EVs, and corresponding to the EV pan-proteome of these strains, were identified (Supplementary Table S2). 160, 164, 168, 171 and 218 proteins were associated with EVs produced by *S. aureus* strains RF122, O11, MW2, O46 and N305, respectively. The majority of these 253 proteins were predicted as cytoplasmic (PRED-LIPO, n = 126; CELLO, n = 118; PSORTb, n = 92) or membrane-associated (PSORTb, n = 80; CELLO, n = 65; PRED-LIPO, n = 67) (Fig. 2A–C). About 10% and 20% of EV proteins are predicted to contain a signal peptide I (SignalP, n = 27; PRED-LIPO, n = 20) and a signal peptide II (SignalP, n = 51; PRED-LIPO, n = 50), respectively (Fig. 2B,D). When considered strain-by-strain, the protein localisation was similar to these overall results. Interestingly, although the predicted signal peptide II-containing proteins (i.e. lipoproteins) represented about 2.5% of the whole proteome of the strains studied here, they accounted for 20% of EV proteomes, which was indicative of their relative enrichment in EVs. In agreement, EV lipoproteins represented about 50% of whole cell lipoproteins. Taken together, these results showed that the predicted subcellular localisation and richness of proteins associated with EVs were markedly similar in the five strains analysed.

### Functional classification of *S. aureus* EV proteomes

The functional characteristics of the 253 *S. aureus* EV proteins were determined using a Clusters of Orthologous Groups (COG) analysis. Overall, *S. aureus* EV-associated proteins were spread over 18 COGs (Supplementary Table S2 and Fig. 2E). Of note, 30% of EV proteins were assigned to the “function unknown” category (n = 49) or not assigned to any COG categories (n = 27). The majority of the remaining proteins (42%; 74/177) could be assigned to COGs related to the general “metabolism” category; *i.e*. energy production and conversion (n = 24, COG C), inorganic ion transport and metabolism (n = 19, COG P), carbohydrate transport and metabolism (n = 9, COG G) and amino acid transport and metabolism (n = 10, COG E). COG J (translation/ribosomal structure and biogenesis) was the most common category, with 38 occurrences. Finally, cell wall/membrane/envelope biogenesis (n = 28, COG M), defence mechanisms (n = 7, COG V) and intracellular trafficking/secretion/vesicular transport (n = 6, COG U) were the most frequent COGs in the general “cellular processes and signalling” category.

Interestingly, 36% (92/253) of the proteins found in EVs could be regarded as virulence-associated proteins (Table 1 and Supplementary Table S3). In particular, these included toxins (e.g. LukMF’ and LukGH leukocidins, α- and β-class phenol soluble modulins, δ-haemolysin), adherence proteins (e.g. elastin binding protein) and host immune evasion factors (e.g. Sbi immunoglobulin-binding protein) as well as proteins involved in antibiotic resistance, survival during pathogenesis and lipoproteins. It should be noted that these factors were among the most abundant proteins in the EV proteome (Supplementary Table S2 and Supplementary Table S3). The distribution of EV proteins according to their functional characteristics was similar between strains, although some subtle differences were observed (Fig. 2E). In summary, the *S. aureus* EVs were rich in virulence-associated factors and contained proteins related to metabolic and translation functions.

**Table 1:** Selection of virulence-associated factors found in EVs from *S. aureus* strains MW2, N305, RF122, O11 and 046.

Function^a^	Label	Gene name	MW2	N305	RF122	O11	O46
**Host immune evasion**							
Immunoglobulin-binding protein	MW2341	sbi	+	+	+	+	+
Bi-component leukocidin LukMF’ subunit M	SAB0782	hlyII	−	−	+	+	−
Bi-component leukocidin LukMF’ subunit F’	SAB0783	lukF	−	−	+	−	−
Delta-hemolysin	MW1959	hld	+	+	+	+	+
Bi-component leukocidin LukGH subunit G	MW1941	lukG	+	+	+	+	+
Bi-component leukocidin LukGH subunit H	MW1942	lukH	+	+	+	+	+
Phenol-soluble modulin alpha 4 peptide	MW0406.1	psmA4	+	+	+	+	+
Phenol-soluble modulin alpha 3 peptide	MW0406.2	psmA3	+	+	+	+	+
Phenol-soluble modulin alpha 2 peptide	MW0406.3	psmA2	+	+	+	+	+
Phenol-soluble modulin alpha 1 peptide	MW0406.4	psmA1	+	+	+	+	+
Beta-class phenol-soluble modulin	MW1056	psmB1	+	+	+	+	+
Beta-class phenol-soluble modulin	MW1057	psmB2	+	+	+	+	+
**Exoenzymes**							
Glyceraldehyde-3-phosphate dehydrogenase	MW0734	gapA1	+	+	+	+	+
Sortase	MW2448	srtA	+	+	−	+	+
**Adhesion and cell wall anchored surface proteins**							
Elastin binding protein	MW1369	ebpS	+	+	+	+	+
Enolase	MW0738	eno	+	+	+	+	+
Extracellular adherence protein Eap/Map	SAO46_01921	map_2	−	−	−	−	+
**Proteins related to biofilm production**							
Thermonuclease	MW1211	nuc	+	+	+	+	+
**Regulatory proteins**							
Accessory gene regulator protein D	SAB1921	agrD	−	−	+	−	−
**Miscellaneous proteins**							
Zinc metalloprotease	MW0466	ftsH	+	+	+	+	+
Serine protease	MW1670	htrA	+	+	+	+	+
Zn-dependent membrane protease	MW1350	yugP	+	+	+	+	+
Bifunctional autolysin	MW0936	atl	+	+	+	+	+
D-alanyl-lipoteichoic acid biosynthesis protein DltD	MW0817	dltD	+	+	+	+	+
Lipoteichoic acid synthase	MW0681	ltaS	−	+	+	−	−
Preprotein translocase subunit SecA	MW0715	secA1	+	+	+	+	+
Preprotein translocase subunit SecDF	MW1587	secDF	+	+	+	+	+

^a^adapted from^84^.

### *S. aureus* EV proteomes reflect strain properties

**Figure 3:**
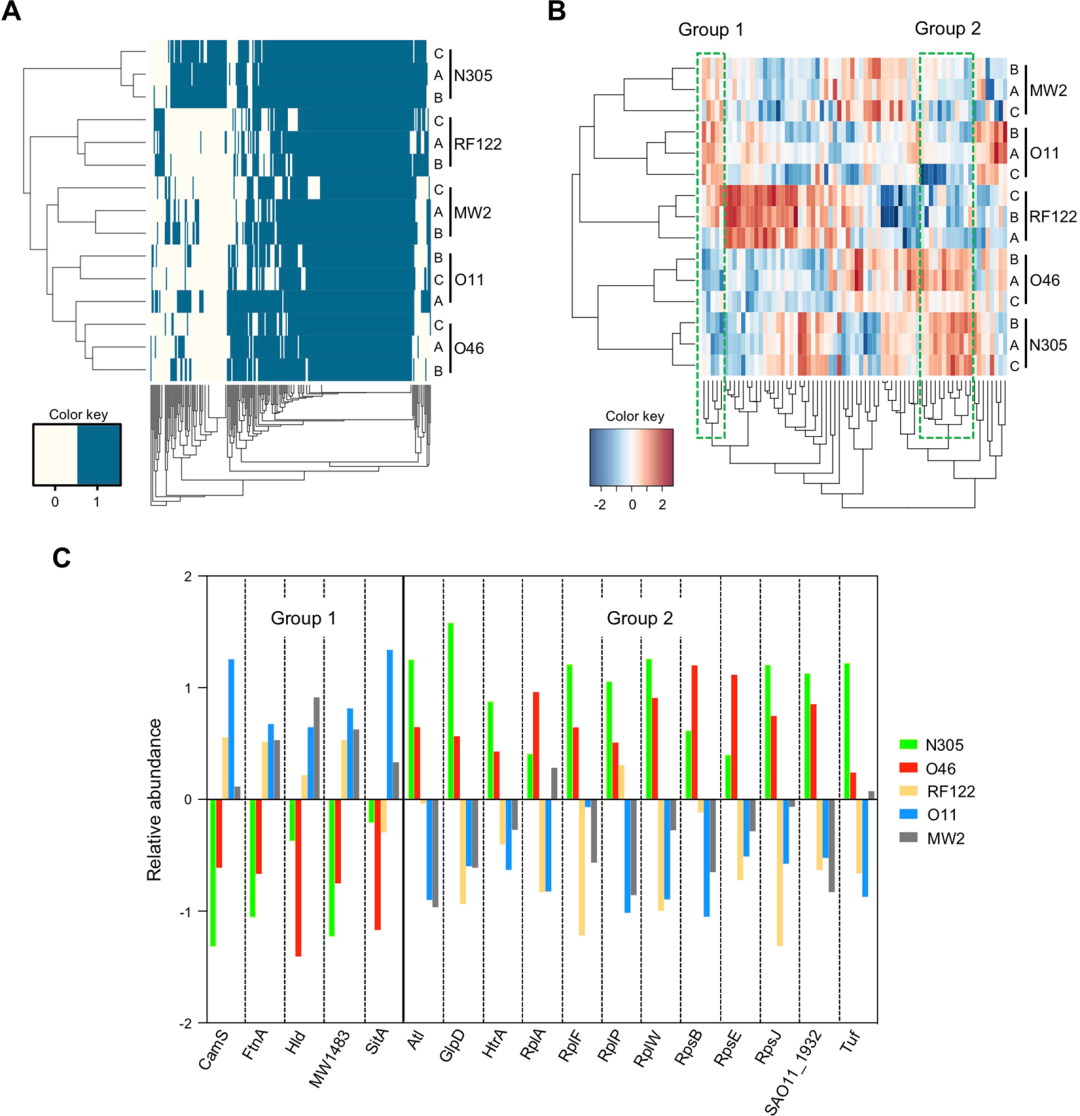
Hierarchical clustering of EV samples. (**A**) Heatmap representation of clustering according to presence/absence of proteins in EV samples. (**B**) Heatmap representation of clustering according to the relative abundance of proteins in EV samples. The group 1 and group 2 proteins with contrasting relative abundance between mild (N305 and O46) and severe (RF122, O11 and MW2) strains are boxed. (**C**) Proteins from group 1 and group 2 and their relative abundance in EVs according to producing strains.

To pinpoint potential correlations between strain properties (*i.e*. host of origin, severity of infection) and the protein cargo found in their EVs, heatmap and clustering analyses were performed on EV proteins from the five *S. aureus* strains (Fig. 3). First at all, whatever the dataset and clustering method, biological triplicates clustered together, suggesting that protein loading into an EV is a reproducible and selective process in each *S. aureus* strain. Based on a matrix for the presence/absence of proteins in vesicles (*i.e*. qualitative analysis; n = 253 proteins), heatmap clustering revealed a partial organisation of EVs according to the host origin of the strains (Fig. 3A). EVs from the two ovine strains formed a group, and MW2 and bovine EVs were the nearest and most distant from this group, respectively.

Based on the relative abundance of proteins in EVs (*i.e*. quantitative analysis; n = 70 proteins), their organisation was different and appeared to reflect the severity of the infections that producing strains could cause (Fig. 3B). Indeed, the first cluster contained EV proteins from strains involved in the development of mild mastitis (N305 and O46), while the second cluster was composed of EV proteins from strains involved in gangrenous and severe mastitis (O11 and RF122, respectively). This cluster also included MW2, a human isolate that is responsible for severe soft tissue and bloodstream infections. Several groups of proteins separated strains as a function of the severity of the infections they cause. Two of these contained proteins with an overall lower (group 1) and higher (group 2) relative abundance of the mild strains N305 and O46 (Fig. 3B,C). Group 1 aggregated virulence-associated factors (δ-haemolysin Hld, CamS, MW1483, FtnA, SitA) whereas group 2 mainly included proteins with housekeeping functions. Taken together, these results showed that the EV proteome reflects strain-specific features.

### Characterisation of a core EV proteome in *S. aureus*

**Figure 4:**
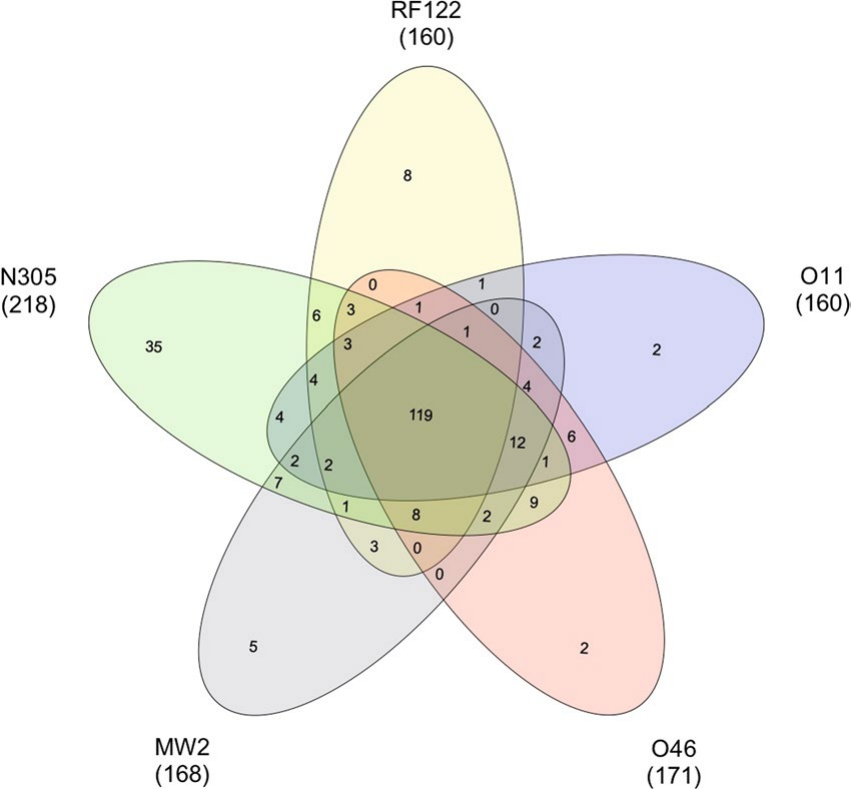
Venn diagram of proteins identified in EVs from *S. aureus* strains MW2, N305, RF122, O11 and O46. The numbers of unique and shared proteins between EVs from several strains are presented.

119 of the 253 EV proteins identified (47%) were common to all strains, while 20% were strain-specific (Fig. 4 and Supplementary Table S2). The core EV proteome, *i.e*. proteins shared by EVs secreted by all the tested strains, was mostly composed of cytoplasmic proteins (ranging from 36% to 60%, depending on the predictor used) and membrane proteins (ranging from 47 to 51%, depending on the predictor used). 54% of EV proteins with a lipoprotein signal peptide present in the dataset belonged to the core EV proteome (n = 27), which also contained more than 80% of proteins belonging to COG F (nucleotide transport and metabolism, 2/2), G (carbohydrate transport and metabolism, 7/7) and O (post-translational modification/protein turnover/chaperones, 4/5) categories. Other major categories concerned COG C (energy production and conversion, 14/24), J (translation/ribosomal structure and biogenesis, 14/38), M (cell wall/membrane/envelope biogenesis, 16/28) and P (inorganic ion transport and metabolism, 12/19). The core EV proteome notably gathered 59% (54/92) of the previously defined virulence-associated proteins (Table 1 and Supplementary Table S3).

To challenge the core EV proteome established for the MW2, N305, RF122, O11 and O46 strains, its overlap with the EV proteomes of Gram-positive bacteria available in the EVpedia database^30^ was analysed (Supplementary Table S4). A large fraction (80%, n = 97) of core EV proteins had homologues present in the EVs of *S. aureus* RN4220. When compared to phylogenetically distant species, proteins belonging to the core EV proteome were also identified, notably in the EVs of *Bacillus subtilis* 168 (n = 43), *Streptococcus pneumoniae* R6 (n = 27), *Streptococcus pyogenes* M1 (n = 38), *Listeria monocytogenes* 10403 S (n = 33), *Mycobacterium tuberculosis* ATCC 25618 (n = 20) and *Clostridium difficile* 630 (n = 25). Nine proteins (the DnaK chaperone protein, GAPDH, ATP synthase subunit beta, Enolase, the Tu elongation factor and four ribosomal proteins) were shared by all of the Gram-positive species analysed.

### Patterns, amino acid composition and physicochemical properties of the *S. aureus* EV proteome

The reproducible presence of proteins associated with EVs in biological replicates, and the characterisation of a core EV proteome shared by strains of different host origins, supported the hypothesis regarding the existence of selective processes for cargo sorting. To address these issues, the presence of protein patterns in core EV proteins, and the physicochemical properties and amino acid and nucleotide composition of their encoding sequences were assessed. The search for local sequence conservation within at least 3 core EV proteins, done by partial local multiple alignment of sequences with Protomata-Learner, enabled the detection of conservated motifs in only 44 out of the 119 non-redundant (<70% identity) core EV proteins. The most prevalent motif was present in 22 protein sequences, while the others were seen in between three and eight sequences (Supplementary Table S5). Among the 9 most significant motifs, five overlapped with four matches of known motifs or domains from PROSITE database: Lipoprotein signal peptide (PS51257), ATP/GTP-binding site motif A (PS00017), the iron siderophore/cobalamin periplasmic-binding domain (PS50983), and the ATP-binding cassette of ABC transporter (PS50893) (Supplementary Fig. S2 and Supplementary Table S5). The four other motifs had no counterparts in the PROSITE and pfam databases. Interestingly, all the motifs detected were composed of charged or aliphatic amino acid residues.

**Figure 5:**
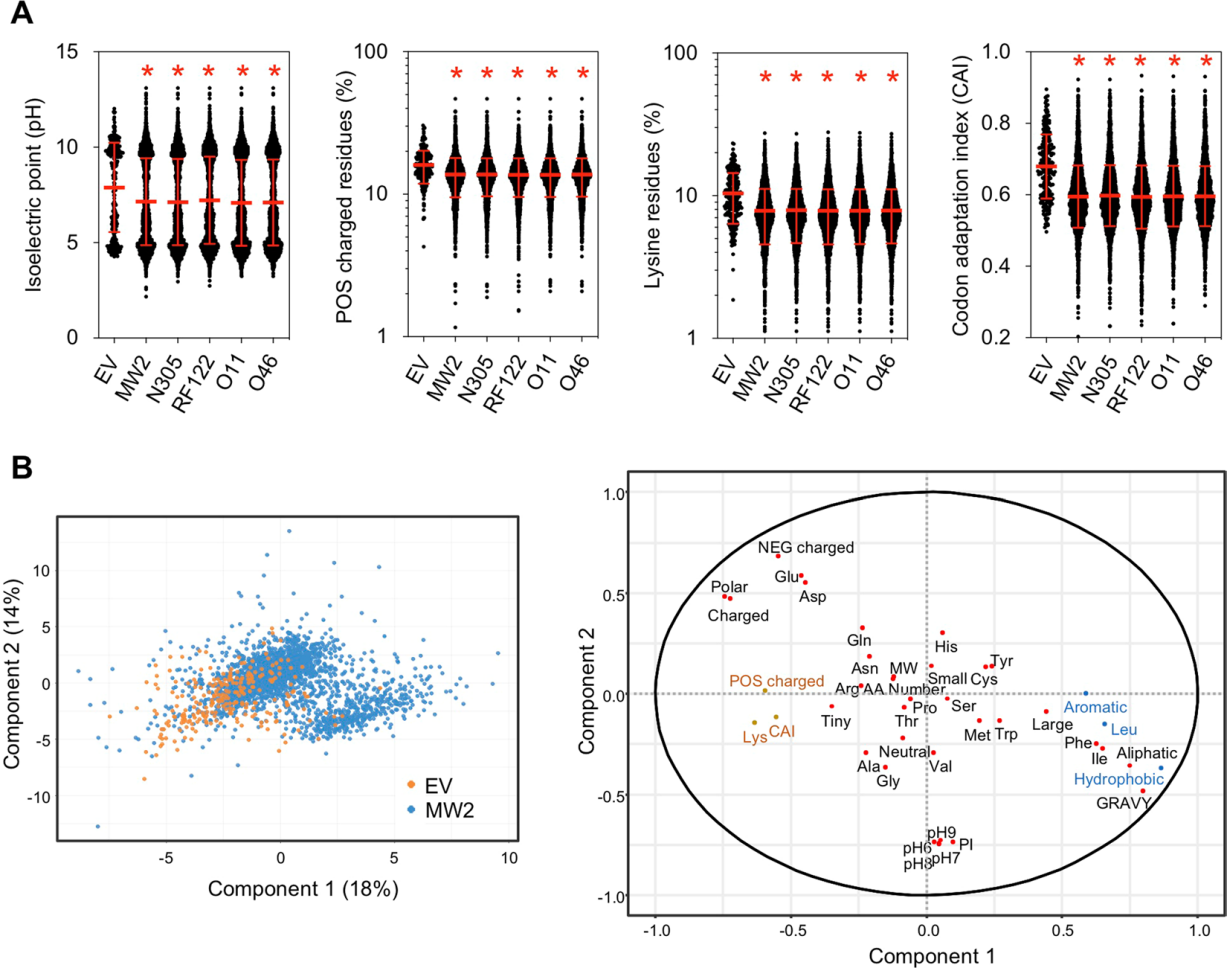
Mean features of EV proteins. (A) Distribution of EV proteins and whole cell proteins of *S. aureus* strains MW2, N305, RF122, O11 and O46 according to their isoelectric point, composition in positively (POS) charged residues, composition in lysine (Lys) residues and the codon adaptation index (CAI) of their corresponding genes. Asterisks indicate statistical significance when compared to the EV group (one-way ANOVA followed by Dunnett’s multiple comparisons test: *P < 0.0001). (B) Score plot and loading plot of the partial least squares-discriminant analysis (PLS–DA) performed on proteins from *Staphylococcus aureus* MW2 in order to differentiate EV proteins (in orange) from proteins not identified in EV proteins (in blue). Variables are the physicochemical properties of proteins, their amino acid composition and the CAI of each of the corresponding genes. In the loading plot, the most significant features positively and negatively associated with EV proteins are indicated in orange and blue, respectively.

The overall physicochemical properties of EV proteins were compared to the whole proteomes of the MW2, N305, RF122, O11 and O46 strains (Supplementary Table S6). These analyses showed that the average isoelectric point, the GRAVY (grand average of hydropathy) value and the net charge at pH 7 of proteins packaged into EVs differed significantly (P < 0.001) from those of the whole cell proteomes (Fig. 5A and Supplementary Fig. S3). Notably, EV proteins were more globular and more positively charged at a physiological pH. In addition, proteins packaged into EVs significantly (P < 0.001) contained more charged, polar and tiny residues and fewer aromatic, aliphatic, hydrophobic and large residues than the whole cell proteome. By contrast, no differences were observed regarding the distribution of proteins according to their number of residues and composition in neutral and small residues, although differences were found at the level of individual amino acids: Ala, Gly and Lys were significantly overrepresented (P < 0.001) in EV proteins, whereas Cys, His, Ile, Leu, Phe and Tyr were underrepresented when compared to whole cell proteomes (Fig. 5A and Supplementary Fig. S4). Finally, a comparison carried out on the Codon Adaptation Index (CAI) of each gene revealed that the CAI values of genes coding for EV proteins were significantly higher (P < 0.0001) compared to those of the whole genome (Fig. 5A). For example, EV proteins in strain MW2 contained 36% more Lys compared to the other cell proteins (10.4% versus 7.6%), and the CAI value of the associated genes was 16% higher (0.68 versus 0.59), although they contained 20% fewer Phe, Ile, and Leu residues.

A discriminant analysis was used to identify the physicochemical properties that best discriminated the proteins according to their detection in EVs or not (Fig. 5B). EV proteins were positively associated with the first axis, which was characterised by charged and polar proteins rich in Glu, Asp and Lys and with high CAI values of the corresponding genes, but were negatively associated with proteins rich in hydrophobic, aliphatic and aromatic amino acids with high GRAVY values.

Overall, these results indicated an absence of conserved motifs within all EV protein sequences, but the existence of specific shared features such as positively charged proteins and high CAI values.

## Discussion

The study of EVs produced by *S. aureus* is an emerging field of research, with pioneering work published in the late 2000s^24^. EVs from this bacterium are certainly some of the best documented among Gram-positive bacteria. They might indeed play a role in *S. aureus* pathogenesis and, as such, they offer interesting perspectives in medical applications. The characterisation of the EV protein cargo has been the subject of particular study^12,17,18,21–24^. However, most of this work focused on clinical isolates with emphasis on the contribution of EVs to the pathogenetic process. Moreover, variations to both the growth conditions used to produce EVs and the procedures for EV preparation and protein identification rarely enabled a reliable comparison of their protein content. Here, we used identical procedures to compare the proteins in EVs. We chose five *S. aureus* strains of diverse host origins (ovine, bovine, human) and causing different types of infection (from mild to severe) to reveal a core EV proteome and gain insight into the mechanisms that drive the selection of proteins to be packaged into EVs. Indeed, understanding how these proteins are selectively packaged offers a possible source of innovation regarding both intervention against pathogenic bacteria and the development of drug delivery systems.

Under our experimental conditions, all these *S. aureus* strains produced and secreted EVs into their surrounding environment. However, some differences between the strains were observed, notably regarding EV production yields and EV size. One remarkable feature was the co-purification of cylindrical (nanotube-like) structures together with EVs in the four animal strain samples. Such nanotubes had previously been observed in Gram-negative and a few Gram-positive bacteria, but with some differences regarding the size of the objects^31^. To the best of our knowledge, ours is the first report to have shown a co-purification of EVs with nanotube-like structures in *S. aureus*. MRSA strains such as MW2 are known to develop a thicker cell wall than methicillin-susceptible *S. aureus* strains^32^. A major barrier to the release of EVs by Gram-positive bacteria is the need to cross a thick cell wall. Whether this accounted for the absence of nanotube-like structures in the MW2 MRSA samples deserves further investigations, as does any biological role they may play.

The EVs of the five *S. aureus* strains studied here displayed similarities in terms of their cargo proteins, and a core EV proteome containing 119 proteins was identified. As previously shown for clinical isolates^12,17,21–24^, *S. aureus* EVs secreted by animal isolates are characterised by the presence of numerous proteins with roles in bacterial survival and pathogenesis. These include toxins (phenol soluble modulins, haemolysin, leukocidin), invasion factors (elastin binding protein, autolysin, extracellular adherence protein), enzymes (nuclease, proteases), surface antigens and immunoglobulin-binding protein. The EV cargo also includes proteins that are involved in metal ion acquisition and allow bacterial cells to circumvent metal ion starvation within the host during infection^33^. No nutritional requirements have yet been described for EVs. Presence of such proteins in the EV proteome thus suggests that EVs act as a source of additional metal ions made available at distance for other invading bacterial cells^34^. Alternatively, they could disturb metal ion acquisition in competing bacterial cells or host cells. The EV cargo also comprises numerous lipoproteins, some of which contribute to the nutrient transport, Toll-like receptor 2 activation and pathogenicity of *S. aureus*^35,36^. Most of these proteins belong to the core EV proteome, indicating that the secretion through EVs of proteins involved in many facets of *S. aureus* pathogenesis represents a common strategy for both animal and human *S. aureus* strains. Moreover, the EVs analysed here also contained antibiotic resistance-associated proteins such as penicillin-binding proteins (in four of the five strains studied) or β-lactamase (in MW2). It was shown that EVs carrying biologically active antibiotic degrading enzymes such as β-lactamases were able to confer transient resistance on susceptible surrounding bacteria^20,37,38^. Indeed, penicillin-binding proteins may act as lures or catching agents and titrate antibiotics in the surrounding medium. Whether these antibiotic resistance-associated proteins do confer transient resistance on distant bacterial cells still needs to be explored and could have interesting implications for the treatment of staphylococcal infections.

A remarkable overlap (>80%) was noted between the core EV proteins that we identified here and the EV proteins previously described in the human *S. aureus* strain RN4220, although we could have expected a fall in the number of shared proteins in the core EV proteome as EVs from more *S. aureus* strains were included. This overlap was even more consistent because different growth conditions and methods for EV preparation and protein identification were used^39^. When compared to EV proteomes from phylogenetically distant Gram-positive bacteria, some proteins also appeared to be conserved. This notably included several moonlighting proteins (e.g. enolase, GAPDH, EF-Tu) with functions in adhesion, membrane fusion, and internalisation^40–43^. Because they are considered as a protein secretion pathway, EVs may thus form part of the mechanism for the sorting of moonlighting proteins^44^. Moreover, conservation of these proteins within the EVs of phylogenetically distant species, including Firmicutes and Actinobacteria, might also account for a generic process involved in intercellular interactions; i.e. the attachment and fusion of EVs to the membrane of targeted cells.

EVs mirror the properties of producing strains despite their compositional and functional similarities so could be considered as potential markers for *S. aureus* infections in both human and veterinary medicine. Indeed, based on their protein content, they can be stratified with respect to the host origin (bovine, ovine, or human) of the strains and the severity of infections they cause. Notably, the relative abundance of proteins in EVs is a criterion that can discriminate them according to the severity of infections. Two groups of proteins with contrasted relative abundances of mild and severe strains were identified during the present study, one of them aggregating several virulence-associated factors (e.g. δ-haemolysin Hld) that was less abundant in mild strains than severe strains. *S. aureus* strains of bovine and ovine origins have evolved to adapt to their animal hosts and differ from human strains at the genotypic and genomic levels^45,46^. In some cases, species-specific traits are also observed at the molecular level, as exemplified for example by the bovine variant of von Willebrand factor-binding protein^47^, or ovine-specific exfoliative toxin type E^48^. Here, none of the EV proteins specifically associated with ovine (n = 6), bovine (n = 6) or human (n = 5) strains presented any functions that could be related specifically to their host origin, with the exception of beta-lactamase for strain MW2. Moreover, among the 52 strain-specific EV proteins identified here (i.e. proteins found in the EVs of one strain) only nine were strictly strain-specific, because the others were present in the predicted whole proteome of the other strains. This demonstrates that drivers other than the presence of a protein in the whole proteome can account for the strain-specific cargo of EV proteins.

The hypothesis that specific and conserved rules for protein loading into EVs exist was further supported by the identification of a core EV proteome for a variety of strains and the partial conservation of protein cargo in phylogenetically distant species. The mechanisms that control protein packaging into EVs still remain obscure. Our analysis has provided some information on drivers that might influence this process. We found compositional biases in the nucleotide sequences of genes encoding EV proteins and in the amino acid sequences of EV proteins themselves. When compared to the whole genome, the CAI value of genes coding for EV proteins was significantly higher. Likewise, EVs contained proteins with more positively charged residues (notably Lys) than in the whole predicted proteome. It is often thought that CAI reflects the level of protein expression, with high CAI values being associated with highly expressed proteins^49–51^. Such a feature might reflect the possible enrichment of highly expressed proteins in EVs. In line with this, almost half of the 50 most abundant whole cytoplasmic proteins of *S. aureus* COL were found in EVs during our study^52^. It seems consistent that the relative abundance of a protein in the whole cell could affect its availability to be packed into EVs: the more abundant a protein, the greater the probability of it being loaded into EVs in the absence of, or in addition to, a selective process. The biases regarding the amino acid composition of EV proteins also suggested the selective packaging of positively charged proteins. In line with this, the theoretical PI value of about half of EV proteins is higher than 8, indicating that they are probably positively charged at a physiological pH. Electrostatic interactions are known to contribute to the subcellular targeting of proteins^53,54^. In particular, the negative charges associated with the inner surface of the membrane can direct cationic and basic proteins to a peripheral membrane localisation^55^. On the other hand, lipid homeostasis of the membrane is a crucial key for EV biogenesis^22,23,56–58^. Notably, an accumulation of negative charges in lipid microdomains contributes to membrane curvature and EV biogenesis. The presence of more negatively charged microdomains at the cytoplasmic surface of the membrane may favour the recruitment of positively charged proteins through electrostatic interactions at the site of EV formation, and hence their selective capture.

Our findings show that EVs act as a “proxy” for producing strains in *S. aureus*, and that host-specific profiles can be seen in the protein composition of EVs as well as in the producing strains. Our analysis of EV protein features suggested that abundance, charge and subcellular localisation could influence the protein availability of the vesicle cargo in *S. aureus*. However, not all proteins with high CAI values, highly expressed or with basic domains were packed within EVs, indicating the existence of a selective process following additional rules. The identification of a core EV proteome offers an opportunity to reveal specific signature(s) (e.g. signals, motifs, domains) in protein sequences that might be characteristic of the molecular machinery (or machineries) dedicated to the selection of proteins into EV cargoes. Despite our efforts, no specific signature could be evidenced in EV protein sequences, except for signal peptide and lipoprotein signal peptide. It is interesting to note that proteins known to be associated with the biogenesis of *S. aureus*-secreted EVs (PSM, autolysin) belong to the core EV proteome. One can suppose that the EV cargo also contains components for the selection of proteins into EVs. Chaperones and protein secretion systems, among others, are good candidates, but they still need to be assessed.

## Methods

### Bacterial strains and growth conditions

*Staphylococcus aureus* strains used in this study were chosen, based on their representativeness with regard to the clonal complex (CC) associated to a given host (Supplementary Table S1). The CC of the strains used in this study are reportedly dominant (or at least frequent) in *S. aureus* strains associated to human (CC1), ovine (CC130), and bovine (CC97, CC151). Furthermore, the studied strains are well documented with genome sequence and phenotype information publicly available (Supplementary Table S1). *S. aureus* ovine strains O11 and O46 were isolated from gangrenous and subclinical mastitis, respectively, and were shown experimentally to induce severe or mild mastitis in ewes^59,60^. Both were isolated in 2003 in Southeast France and kindly provided by Dr Eric Vautor (ANSES, Sophia Antipolis, France). Bovine strain RF122 was isolated from a case of clinical mastitis^61,62^. Bovine strain Newbould 305 (N305) was shown to reproducibly induce mild and chronic mastitis in bovines^63,64^. RF122 and N305 were kindly provided by Pr Ross J. Fitgerald (Edinburgh Infectious Diseases, Roslin Institute, University of Edinburgh, UK) and Dr Pascal Rainard (ISP, INRAE, Nouzilly France), respectively. The human strain MW2 (ATCC BAA-1707) was isolated from a severe case of hospital acquired infection^65^ and is reportedly highly virulent^66^. The human strain MW2 was obtained from the Laboratory of Human Bacterial Pathogenesis, National Institutes of Health (Bethesda, MD, USA). The *S. aureus* strains were grown in Brain Heart Infusion (BHI) medium (Difco, pH 7.4) at 37 °C under agitation (150 rpm). The concentrations and growth phases of the bacteria were estimated from spectrophotometric measurements of optical density at 600 nm (VWR V-1200 spectrophotometer). They were further confirmed routinely by counting the colony forming units (CFU) on BHI agar using the micromethod^67^.

### Purification of *S. aureus*-secreted EVs from culture supernatants

EVs were purified from *S. aureus* culture supernatants as described previously^18^. Briefly, sub-cultured cells at the end of the exponential phase were diluted in 1 L of fresh BHI medium. The cultures were grown until the early stationary phase to optimise the number of EVs that could be recovered. The cells were then pelleted at 6,000 *g* for 15 min and the supernatant fraction was filtered through a 0.22 µm vacuum filter (PES). The filtrate was concentrated using the Amicon ultrafiltration system (Millipore) with a 100 kDa filter and subjected to ultracentrifugation at 150,000 *g* for 120 min at 4 °C. The pellet was then re-suspended in 8% sucrose in tris-buffered saline (TBS) (150 mM NaCl, 50 mM Tris-Cl, pH 7.5), overlaid with sucrose dilutions ranging from 8% to 68% in TBS and centrifuged at 100,000 *g* for 150 min at 4 °C in a SW 55 Ti rotor (Beckman Coulter). The different density fractions were collected, and those containing a similar number of EVs, with a similar particle-size distribution and protein pattern were pooled, centrifuged at 150,000 *g* for 120 min at 4 °C and re-suspended in TBS. EV fractions and isolated EV samples were routinely verified by electron microscopy and Bradford assay (Bio-Rad) and quantified using Nanoparticle Tracking Analysis before being stored at −20 °C until use.

### Electron microscopy

The purity and quality of EV samples were confirmed by negative staining electron microscopy and cryo-electron tomography, as previously described^18^. Images were acquired and analysed at the Rennes Microscopy Imaging Centre platform (MRic MET) (University of Rennes 1, Rennes, France).

### Nanoparticle tracking analysis (NTA)

NanoSight NS300 (Malvern Instruments, United Kingdom) with a 488 nm laser module and sCMOS camera type were used for all measurements of *S. aureus* EVs. All counts were performed in replicates of five videos of 60 s for each sample (n = 3) and measured in flow mode using a syringe pump. The EVs were thawed and diluted in TBS until optimum visualisation of a maximum number of vesicles was achieved. All quantifications were performed at a controlled temperature of 25 °C, and the measurement data captured were analysed using NTA 3.3 software (Malvern Instruments). We confirmed that the TBS was free of contamination with any other nanoparticles prior to all measurements.

### Identification of proteins in *S. aureus* EVs

Three independent biological replicates of each *S. aureus* purified EV were digested for NanoLC-ESI-MS/MS analysis. Approximately 50 µg of EVs were pelleted at 150,000 *g* for 2 h at 4 °C and suspended in a solution of 6 M Guanidine-HCl (Sigma-Aldrich), 50 mM Tris-HCl (pH 8.0) (VWR) and 2 mM DTT (Sigma-Aldrich). The EVs were heated at 95 °C for 20 min and cooled in 50 mM NH_4_HCO_3_ (pH 7.8) (Sigma-Aldrich). The samples were then digested in solution using sequencing grade-modified trypsin (Promega) at an enzyme:protein ratio of 1:50 for 15 h at 37 °C, as previously reported^24^. After digestion, the peptides were stored at −20 °C until further analysis. NanoLC-MS/MS experiments were performed as previously described^18^. The peptides were identified from the MS/MS spectra using X!TandemPipeline software^68^ and searches performed against the genome sequence of *S. aureus* MW2, N305, RF122, O11 and O46 strains. The database search parameters were specified as follows: trypsin cleavage was used and the peptide mass tolerance was set at 10 ppm for MS and 0.05 Da for MS/MS. Methionine oxidation was selected as a variable modification. For each peptide identified, a minimum e-value lower than 0.05 was considered to be a prerequisite for validation. A minimum of two peptides per protein was imposed, resulting in a false discovery rate (FDR) < 01% for peptide and protein identifications.

For protein quantification, each peptide identified by tandem mass spectrometry was quantified using the free MassChroQ software^69,70^ before data treatment and statistical analysis with R software (R 3.2.2, Project for statistical computing). A specific R package called ‘MassChroqR’ (v0.4.3) was used to automatically filter dubious peptides and group the peptide quantification data into proteins. Two different and complementary analytical methods were used, based on peak counting or XIC (eXtracted Ion Current). For peak counting, variance analysis was performed on proteins with a minimum peak ratio of 1.5 between both culture conditions. Proteins with an adjusted P-value < 0.05 were considered to be significantly different. For XIC based quantifications, normalisation was performed to take account of possible global quantitative variations between LC-MS runs. Peptides shared between different proteins were automatically excluded from the dataset, as were peptides present in fewer than two of the three biological replicates. Missing data were then imputed from a linear regression based on other peptide intensities for the same protein. Analysis of variance was used to determine proteins whose abundance differed significantly between strains.

### Bioinformatics analysis

All proteins were searched in the NCBI (https://www.ncbi.nlm.nih.gov/) and UniProt (http://www.uniprot.org/) databases. In-house bash script embedding NCBI’s Entrez Direct tools was used to retrieve locus tags from Uniprot id, and PATRIC^71^ was used to find locus tags of orthologous genes between the five strains. Several pipelines based on different methods were used to predict EV protein localisations: CELLO^72^, PredLipo^73^, PsortB version 3.0.2^74^ and SignalP 5.0^75^. Clusters of Orthologous Groups of proteins (COGs) were used to categorise *S. aureus* EV proteins^76^. Motifs in protein sequences were discovered using Protomata learner 2.0 (http://tools.genouest.org/tools/protomata/learn/)^77^, while matches of known PROSITE motifs and profiles were identified with ScanProsite^78^. Amino acid composition, physicochemical properties, the GRAVY (grand average of hydropathy) value and the theoretical isolectric point of proteins from EVs and whole cells in the N305, RF122, O11, O46 and MW2 strains were computed with COPid^79^ and the Sequence Manipulation Suite^80^ from genomic data present in the MicroScope platform^81^. The Codon Adaptation Index (CAI) values of each gene were obtained from the MicroScope platform. Moonlighting proteins were identified using the MoonProt database^82^. Comparisons of proteins from the EVs of *S. aureus* strains was performed using InteractiVenn^83^.

### Statistical analyses

The data were presented as mean ± standard error. The differences between groups were verified using one-way ANOVA followed by Dunnett’s or Tukey’s multiple comparisons test with GraphPad Prism version 8.3.1 for macOS (GraphPad Software, San Diego, California USA). A P-value lower than 0.01 was considered to be significant. A partial least squares discriminant analysis (PLS–DA) was performed using the mixOmics R package to identify the variables that best discriminated the MW2 cell proteins identified in EVs from the other MW2 proteins among the 40 variables that described the amino acid composition and physicochemical properties of the proteins, and the CAI of the corresponding genes. PLS–DA was performed on a dataset made up of 40 variables for the 2759 proteins in *S. aureus* MW2, including 253 EV proteins and 2506 proteins not identified in EVs.

## Supplementary information

Supplementary Figures 1-4.

Supplementary Tables 1-6.

## Data Availability

Raw data are available at this adress: 10.15454/SMFFWK.
