# Supplementary material for: Extracellular vesicles produced by human and animal Staphylococcus aureus strains share a highly conserved core proteome
Source: Sci Rep. 2020 May 21;10:8467. doi: 10.1038/s41598-020-64952-y (PMC7242376; doi:10.1038/s41598-020-64952-y)
Supplement: Supplementary file 1 — Supplementary Figures 1-4. [file 41598_2020_64952_MOESM1_ESM.docx]

**Supplementary Information**

**Extracellular vesicles produced by human and animal *Staphylococcus aureus* strains share a highly conserved core proteome**

Natayme Rocha Tartaglia^1,2^, Aurélie Nicolas^1^, Vinícius de Rezende Rodovalho^1,2^, Brenda Silva Rosa da Luz^1,2^, Valérie Briard-Bion^1^, Zuzana Krupova^3^, Anne Thierry^1^, François Coste^4^, Agnes Burel^5^, Patrice Martin^6^, Julien Jardin^1^, Vasco Azevedo^2^, Yves Le Loir^1^, Eric Guedon^1,*^

# ^1^ INRAE, Agrocampus Ouest, STLO, F-35000, Rennes, France

# ^2^ Federal University of Minas Gerais, Belo Horizonte, MG, Brazil

# ^3^ Excilone, F-78990, Elancourt, France

^4^ Univ Rennes, Inria, CNRS, IRISA, Rennes, France

^5^ Univ Rennes, CNRS, Inserm, BIOSIT - UMS 3480, US_S 018, F-35000 Rennes, France

^6^ INRAE, Université Paris-Saclay, AgroParisTech, UMR GABI, F-78350, Jouy-en-Josas, France

* Corresponding author’s e-mail: eric.guedon@inra.fr


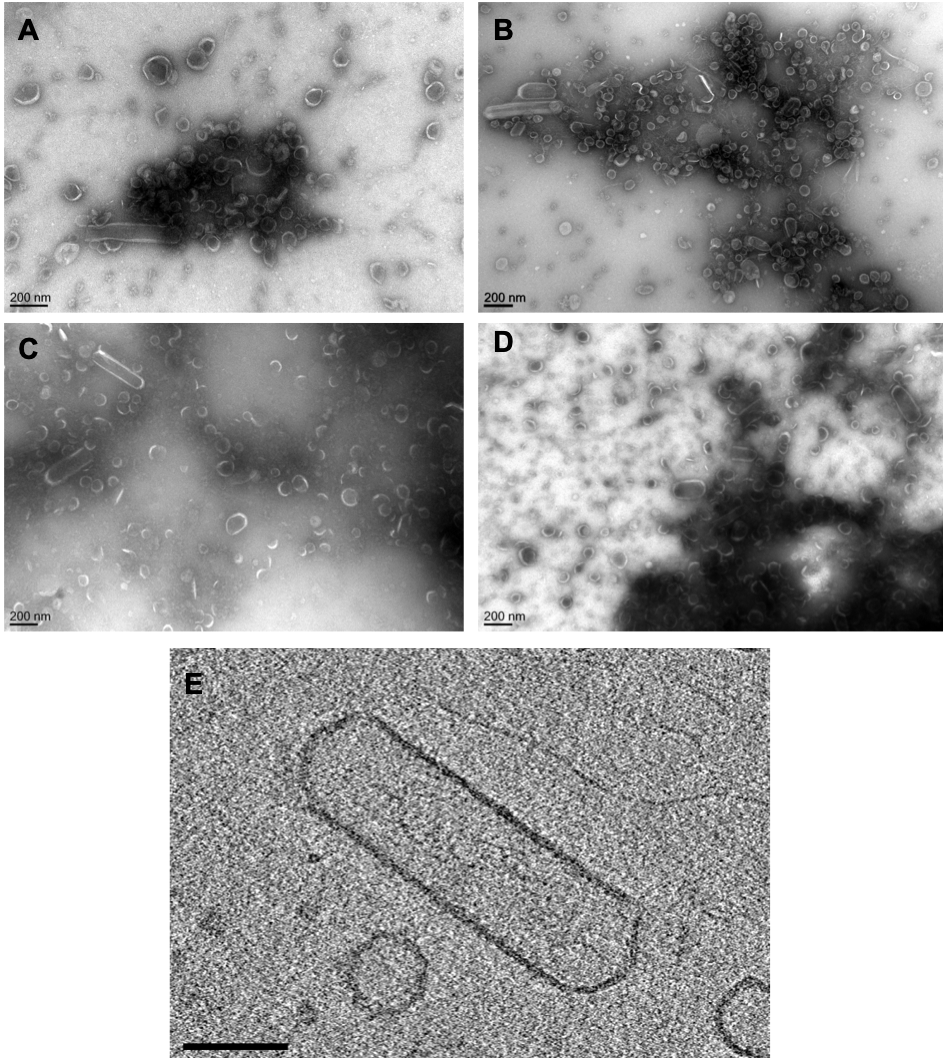


**Figure S1.** Nanotube-like structures co-purified with *S. aureus* EVs. (A-D) Representative electron microscopic images of negatively stained EVs purified from culture supernatant of *S. aureus* strains N305 (A), RF122 (B), O11 (C) and O46 (D).(E) Slice through a cryo-electron tomogram obtained from *S. aureus* N305 EVs with a nanotube-like structure. Scale bar = 100 nm.


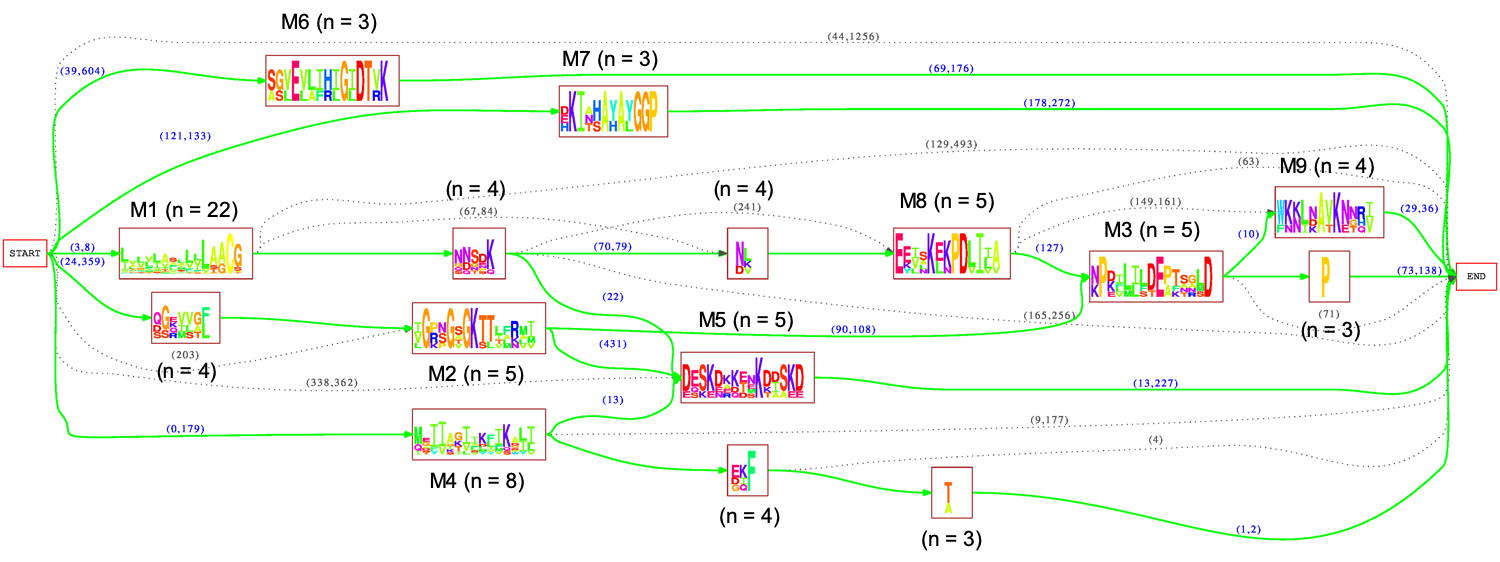


**Figure S2.** Protomaton built from non-redundant set of core EV protein sequences with 9 most significant motifs (length > 10) labeled from M1 to M9 and number n of sequences involved in each motif. Non-redundant set  (built with MMseqs2^1^, < 70% identity) contained all core EV protein sequences except A0A0H3K2S2. This set was given as input to Protomata v2.0 (1813) through <http://tools.genouest.org/tools/protomata> webserver with default PLMA parameters (minimum and maximum size of fragments of 1 and 15, weak consensus) except the similarity threshold lowered to 2 to enable observing weaker similarities. A quorum of 3 unweighted sequences by motif and no pseudocounts were used to build the protomaton.

1. Mirdita M, Steinegger M and Soeding J. MMseqs2 desktop and local web server app for fast, interactive sequence searches. Bioinformatics, doi: 10.1093/bioinformatics/bty1057 (2019).


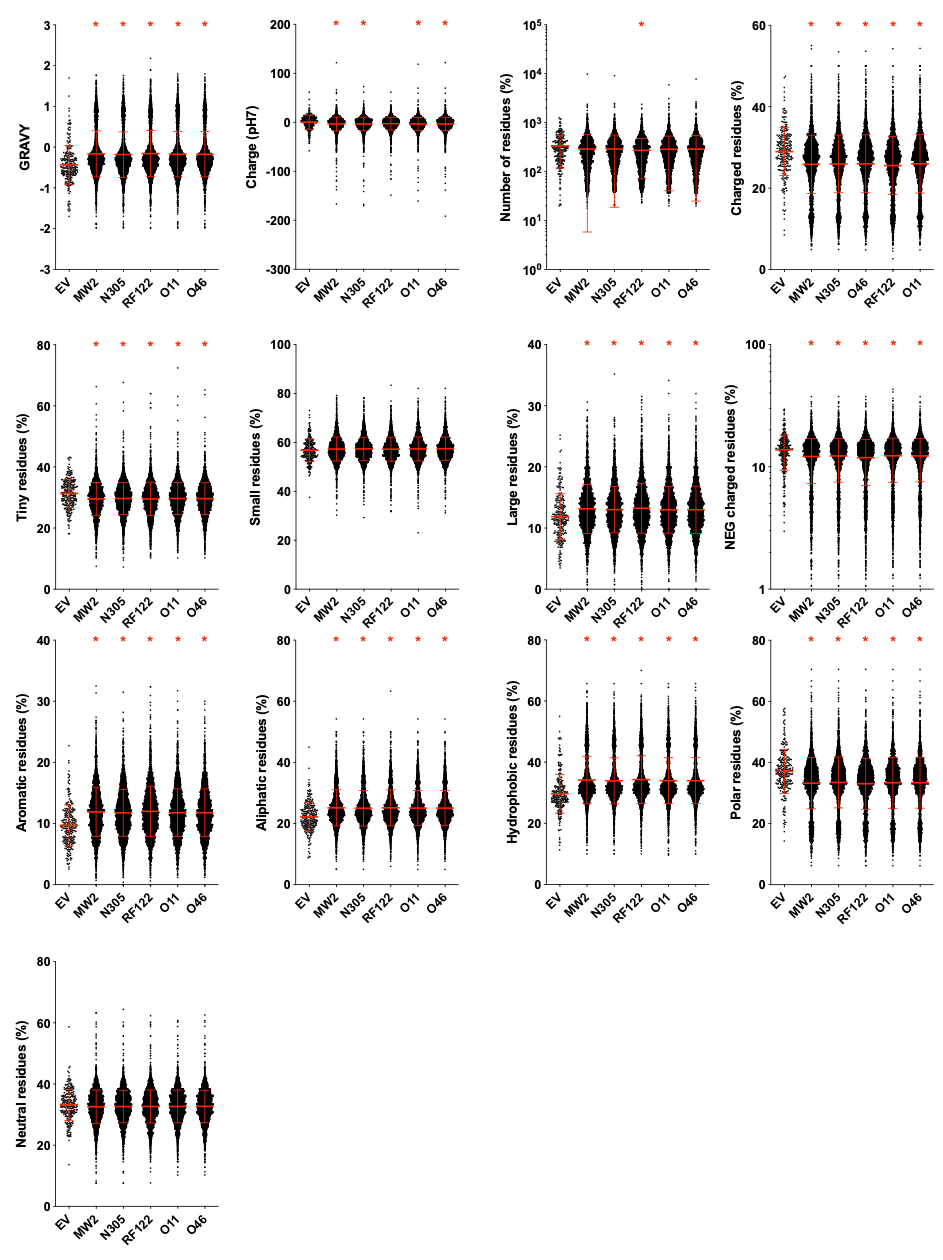


**Figure S3.** Comparison of the physicochemical properties of proteins found in EVs *versus* whole cell of *Staphylococcus aureus* strains MW2, N305, RF122, O11 and O46. The isoelectric point, the GRAVY value and the charge Physicochemical composition of proteins were computed with Sequence Manipulation Suite (https://www.bioinformatics.org/sms2/) and COPID (<https://webs.iiitd.edu.in/raghava/COPid/index.html>). Charged residues: DEKHR. Positively (POS) charged residues: KRH. Negatively (NEG) charged residues: DE. Aliphatic residues: ILV. Aromatic residues: FHWY. Polar residues: DERKQN. Neutral residues: AGHPSTY. Hydrophobic residues: CFILMVW. Tiny residues: ACDGST. Small residues: EHILKMNPQV. Large residues: FRWY. Asterisks indicate statistical significance when compared to the EV group (one-way ANOVA followed by Dunnett’s multiple comparisons test: *, P <0.001).


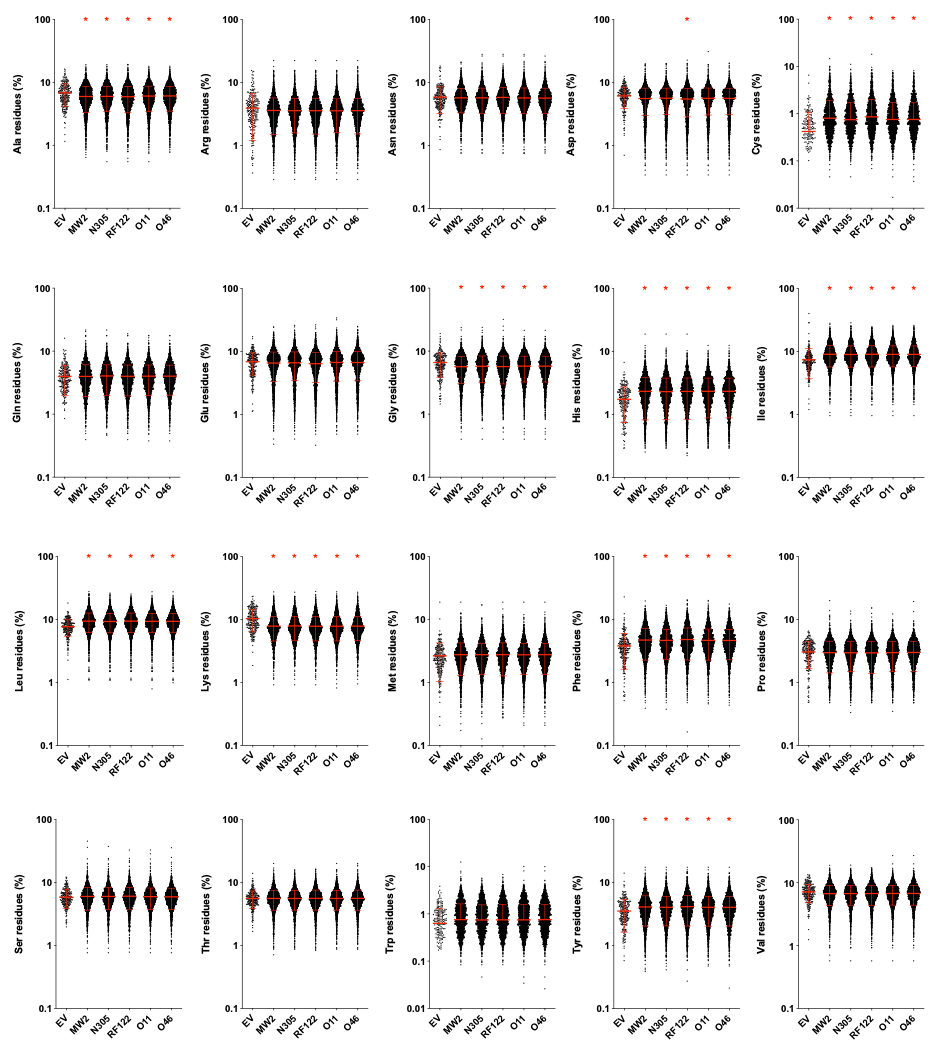


**Figure S4.** Comparison of amino acid composition of proteins found in EVs *versus* whole cell of *S. aureus* strains MW2, N305, RF122, O11 and O46. Amino acid of proteins were calculated from the COPID server (<https://webs.iiitd.edu.in/raghava/COPid/index.html>). Asterisks indicate statistical significance when compared to the EV group (one-way ANOVA followed by Dunnett’s multiple comparisons test: *, P <0.001).
